# Effect of low-intensity pulsed ultrasound on distraction osteogenesis: a systematic review and meta-analysis of randomized controlled trials

**DOI:** 10.1186/s13018-018-0907-x

**Published:** 2018-08-17

**Authors:** Shenghan Lou, Houchen Lv, Zhirui Li, Peifu Tang, Yansong Wang

**Affiliations:** 1 Department of Spine Surgery, The First Affiliated Hospital of Harbin Medical University, No. 23 Youzheng Road, Harbin, 150001 Heilongjiang People’s Republic of China; 2 Department of Orthopedics, Chinese PLA General Hospital, No. 28 Fuxing Road, Beijing, 100853 People’s Republic of China

**Keywords:** Low-intensity pulsed ultrasound, Distraction osteogenesis, Fracture healing, Meta-analysis

## Abstract

**Background:**

Low-intensity pulsed ultrasound (LIPUS) is a common adjunct used to promote bone healing for fresh fractures and non-unions, but its efficacy for bone distraction osteogenesis remains uncertain. This study aims to determine whether LIPUS can effectively and safely reduce the associated treatment time for patients undergoing distraction osteogenesis.

**Methods:**

MEDLINE, EMBASE, and the Cochrane Library were searched until May 1, 2018, without language restriction. Studies should be randomized controlled trials (RCTs) or quasi-RCTs of LIPUS compared with sham devices or no devices in patients who undergo distraction osteogenesis. The primary outcome was the treatment time. The secondary outcome was the risk of complications. Treatment effects were assessed using mean differences, standardized mean differences, or risk ratios using a random-effects model. The Cochrane risk-of-bias tool was used to assess the risk of bias. The *I*^2^ statistic was used to assess the heterogeneity. The GRADE system was used to evaluate the evidence quality.

**Results:**

A total of 7 trials with 172 patients were included. The pooled results suggested that during the process of distraction osteogenesis, LIPUS therapy did not show a statistically significant reduction in the treatment time (mean difference, − 8.75 days/cm; 95% CI, − 20.68 to 3.18 days/cm; *P* = 0.15; *I*^2^ = 72%) or in the risk of complications (risk ratio, 0.90 in favor of LIPUS; 95% CI, 0.65 to 1.24; *I*^2^ = 0%). Also, LIPUS therapy did not show a significant effect on the radiological gap fill area (standardized mean difference, 0.48 in favor of control; 95%CI, − 1.49 to 0.52; *I*^2^ = 0%), the histological gap fill length (standardized mean difference, 0.76 in favor of control; 95%CI, − 1.78 to 0.27; *I*^2^ = 0%), or the bone density increase (standardized mean difference, 0.43 in favor of LIPUS; 95%CI, − 0.02 to 0.88; *I*^2^ = 0%).

**Conclusions:**

Among patients undergoing distraction osteogenesis, neither the treatment time nor the risk of complications could be reduced by LIPUS therapy. The currently available evidence is insufficient to support the routine use of this intervention in clinical practice.

**Trial registration:**

CRD 42017073596

**Electronic supplementary material:**

The online version of this article (10.1186/s13018-018-0907-x) contains supplementary material, which is available to authorized users.

## Background

Bone loss represents a complex set of challenges in terms of treatment and functional recovery, and management of bone defects is a challenging procedure in orthopedic surgery [1]. Autologous bone grafting along with soft tissue surgical reconstruction has been advocated for large bone defects [2]. However, there are several limitations and complications associated with this treatment, including an insufficient amount of autologous bone available for reconstruction, unavailability of autologous bone in growing children, and donor site morbidity [3, 4].

Distraction osteogenesis, developed by Ilizarov [5, 6], is a technique that creates new bone formation between opposing bone segments at the osteotomy site and activates regeneration of the soft tissue matrix surrounding the hard tissue. It overcomes the complications and limitations associated with bone grafting, providing a reliable alternative technique for the treatment of bone defects [7, 8]. Although distraction osteogenesis has a clear benefit for patients with not only skeletal defect but also any malalignment, shortening, soft tissue loss, or joint contractures, it is unfortunately associated with many complications, such as pin tract infections, soft tissue contractures, refractures, and pseudoarthrosis [8–10]. The prolonged duration of treatment is one of the major drawbacks of distraction osteogenesis, which is the primary cause for the above complications. In addition, since the length of time taken for bone union is a key factor in the patient’s recovery [11], the prolonged treatment time is also harmful for the patient’s recovery. Shortening the treatment time can make the technique more safe and cost-effective.

Low-intensity pulsed ultrasound (LIPUS) can cause pressure waves, converting to a biochemical signal inside the cells; stimulating signal transduction, blood flow, and angiogenesis; and promoting protein synthesis, calcium uptake, and osteogenic gene expression [12, 13]. LIPUS appears to be an effective non-invasive adjunctive therapy to promote the bone healing process, which has been approved by the US Food and Drug Administration and the National Institute of Clinical Excellence for the treatment of fresh fractures, delayed unions, and non-unions [14]. It is thought that the application of LIPUS during distraction osteogenesis can promote bone healing, reduce the treatment time, and thereby improve patient’s recovery. However, the results are not totally convincing or consistent among all trials. Some trials reported a positive effect of LIPUS during the process of distraction osteogenesis [15–19], but some trials did not confirm this positive effect [20–24].

Previous systematic reviews analyzing the effect of LIPUS on fracture healing casually noted this potential [25–28], and a comprehensive meta-analysis on the topic has been available to date [29]. This meta-analysis suggests that LIPUS therapy may provide a reduction in the overall treatment time for tibial distraction osteogenesis [29]. However, owing to the limited sample sizes and the high risk of bias of the included trials, the author said that the conclusion should be considered with caution [29]. It also should be noted that this meta-analysis only focused on radiographic healing over other patient-important outcomes, such as bone density increases and the incident rate of complications. In addition, after that meta-analysis [29], a recently published trial, by far the largest trial on LIPUS treatment for distraction osteogenesis, determined that LIPUS did not influence the rate of bone healing in patients who undergo distraction osteogenesis [22].

Thus, an updated meta-analysis is necessary to provide a high-quality evidence for the use of LIPUS in patients who undergo distraction osteogenesis. The purpose of this meta-analysis of randomized controlled trials (RCTs), comparing the different effects between LIPUS treatment and sham devices or no devices, is to determine whether LIPUS can (1) reduce the associated treatment time, (2) reduce the incident rate of complications, and (3) improve bone regeneration and bone density for patients undergoing long-bone distraction osteogenesis.

## Methods

This systematic review was reported according to the Preferred Reporting Item for Systematic Review and Meta-Analysis checklist [30]. A formal protocol was developed and registered on the PROSPERO international prospective register of systematic reviews (prospectively registered, CRD42017073596. Registered 11 February 2018. Https://www.crd.york.ac.uk/prospero/display_record.php?RecordID=73596).

### Search strategy

MEDLINE, EMBASE, and the Cochrane Library were searched from their inception until May 1, 2018, without language restriction by two independent authors (SHL and HCL). Additionally, reference lists from retrieved trials, reports, conference abstracts, and reviews were manually scanned to further identify potentially eligible trials. The search strategy was developed using relevant text words as well as Medical Subject Headings that consisted of terms relevant to “Distraction Osteogenesis,” “ultrasonic therapy,” “ultrasonography,” and “randomized control trial” (for the detailed search strategy, see Additional file 1: File S1).

### Eligibility criteria

All RCTs of any duration assessing the association of LIPUS compared with placebo (or no additional treatment) among adults (aged ≥ 18 years) of any sex undergoing treatment with distraction osteogenesis regardless of location (long bone, short bone, flat bone, or irregular bone) of the body were potentially eligible for inclusion.

### Outcome

The primary outcome was the reduction of treatment time during distraction osteogenesis, as measured by the bone healing index [31, 32], which is the time to maturation of the regenerate by the size of distraction gap, expressed in days per centimeter. The secondary outcome was the risk of complications. In addition, any other outcomes used to assess the time effect of LIPUS on bone healing were considered.

### Study selection

Our search records were imported into ENDNOTE X7 reference management software, and the duplicate records were removed both electronically and manually. After excluding the duplicate and apparently irrelevant articles, the remaining studies were further reviewed by reading the full text to assess the eligibility for inclusion. Titles, abstracts, and full-text articles were screened independently by two authors (SHL and HCL) for eligibility, with discrepancies discussed with a third author (ZRL).

### Data extraction

Information was carefully extracted from all the eligible publications independently by two independent reviewers (SHL and HCL), and disagreements were resolved through discussion or by seeking an independent third author (ZRL). A standard data extraction form was created using Microsoft Excel 2016 to collect data of interest. The major categories of variables to be coded were (1) study characteristics, (2) participant characteristics, (3) type of intervention (type, dose, duration), and (4) outcome characteristics. When data were only presented graphically, GetData Graph Digitizer 2.26 software was used to digitize and extract the data. When the original data were not available, we calculated the data through the available coefficients. For example, we computed the mean from median and the standard deviation (SD) from standard error (SE), interquartile range (IQR), or *P* values, according to the methods described in the Cochrane Handbook [33].

### Risk of bias assessment

Two authors (SHL and HCL) independently assessed the risk of bias using the Cochrane risk-of-bias tool [34]. Bias was assessed across the following seven domains: (1) random sequence generation (selection bias), (2) allocation concealment (selection bias), (3) blinding of participants and personnel (performance bias), (4) blinding of outcome assessment (detection bias), (5) incomplete outcome data (attrition bias), (6) selective reporting (reporting bias), and (7) other biases. Each aspect could further be classified as a low, high, or unclear risk. Any disagreements were resolved through discussion and sometimes with another reviewer (ZRL) if necessary.

### Data synthesis and analysis

The dichotomous outcomes are expressed as the risk ratios (RRs) and the 95% confidence interval (CI), using the Mantel-Haenszel method. The continuous outcomes are expressed as the mean differences (WMDs) or the standardized mean differences (SMDs) with their 95% CI, using the generic inverse variance methods.

Random-effects meta-analyses were conducted using the DerSimonian-Laird method [35], which provided more conservative estimated effects. To assess heterogeneity in results of individual studies, we used the *I*^2^ statistic (0–40%, not important; 30–60%, moderate heterogeneity; 75–100%, considerable heterogeneity) [36]. Publication bias was assessed using the Egger regression test [37], if at least 10 trials were included in a meta-analysis, for funnel asymmetry in addition to visual inspection of the funnel plots.

When there was a significant heterogeneity (*I*^2^ > 50%) [33], both sensitivity analyses and subgroup analyses were performed to explore possible sources of heterogeneity. Sensitivity analyses were conducted using sequential omission of a single study from the total studies to evaluate the influence of each study on the pooled effect estimates. Subgroup analyses were performed based on the overall risk of bias (“low risk of bias” versus “high risk of bias”).

A two-sided *P* value of less than or equal to .05 was deemed statistically significant. All analyses were conducted in Review Manager (version 5.3) and Comprehensive Meta-Analysis (version 2.0).

### Quality of evidence

The quality of the evidence was assessed according to using the Grading of Recommendations Assessment, Development, and Evaluation (GRADE) guidelines, which uses the domains of risk of bias, inconsistency, indirectness, imprecision, and publication bias in results [38]. Each assessment result was rated as very low, low, moderate, or high. Summary tables were constructed using the GRADE Profiler (version 3.6).

## Results

### Study selection

**Fig. 1:**
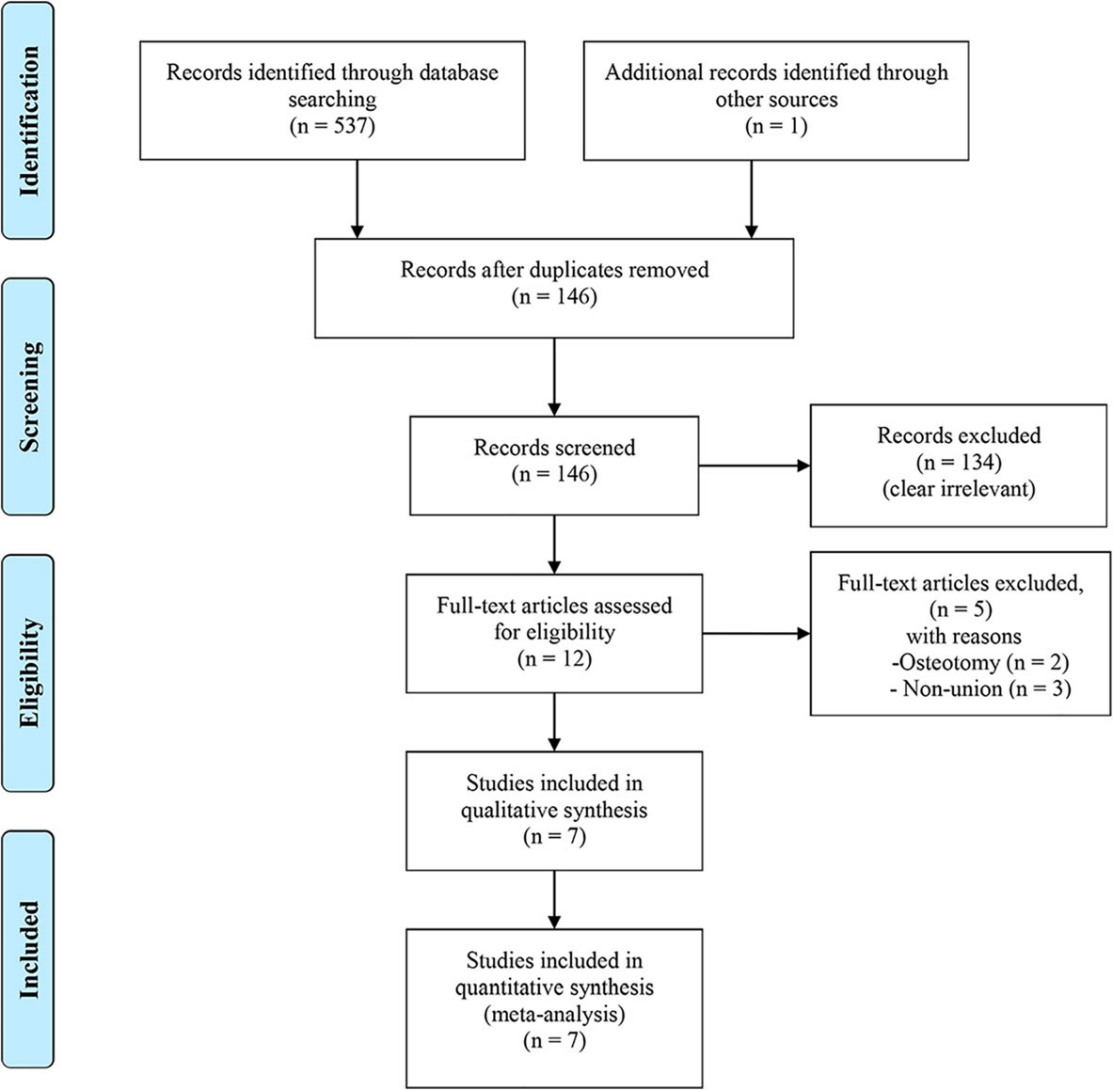
Flow diagram shows the process of literature selection

Figure 1 presented the process of literature selection for this meta-analysis. A total of 538 articles were obtained through electronic and hand searches. After 392 duplicates were removed, the titles and abstracts of 146 records were reviewed, 134 records were excluded for not meeting the inclusion criteria, and, thus, the remaining 12 articles were retrieved for further assessment. Two trials were excluded because the osteotomy was treated without distraction osteogenesis [39, 40]. Three trials were excluded because the non-union was treated without distraction osteogenesis [41–43]. Finally, 7 trials fulfilled our inclusion criteria and were included in our meta-analysis [15, 18–22, 44].

### Study characteristics

The study characteristics of the included trials are summarized in Table 1. All the 7 included trials were RCTs, published between 2004 and 2017 [15, 18–22, 44]. The sample sizes ranged from 8 to 62, with a total of 172 patients. Five trials performed distraction on the tibia [15, 18, 19, 22, 44], and 2 performed distraction on the mandible [20, 21]. The LIPUS treatment was used for 20 min every day for all the included trials.

**Table 1:** Summary of study characteristics of included trials

Study	Sample size		Bonelocation	Mean age	Mean distraction gap (cm)	Distraction rate (mm/day)	LIPUS duration	LIPUS dose (min/day)
	LIPUS	Control						
Tsumaki N [44]	21	21	Tibia	68	0.5	1	Until healing	20
El-Mowafi HM [18]	10	10	Tibia	35	6.1	1	Until healing	20
Schortinghuis J [21]	4	4	Mandible	65	0.66	1	5 weeks	20
Schortinghuis J [20]	5	4	Mandible	56	0.51	1	7 weeks	20
Dudda M [15]	16	20	Tibia	39	6.6	Unclear	Until healing	20
Salem KH [19]	12	9	Tibia	30	7.9	1	Until healing	20
Simpson AH [22]	32	30	Tibia	37	4. cm	0.75	Until healing	20

*LIPUS* low-intensity pulsed ultrasound

### Risk of bias assessment

**Fig. 2:**
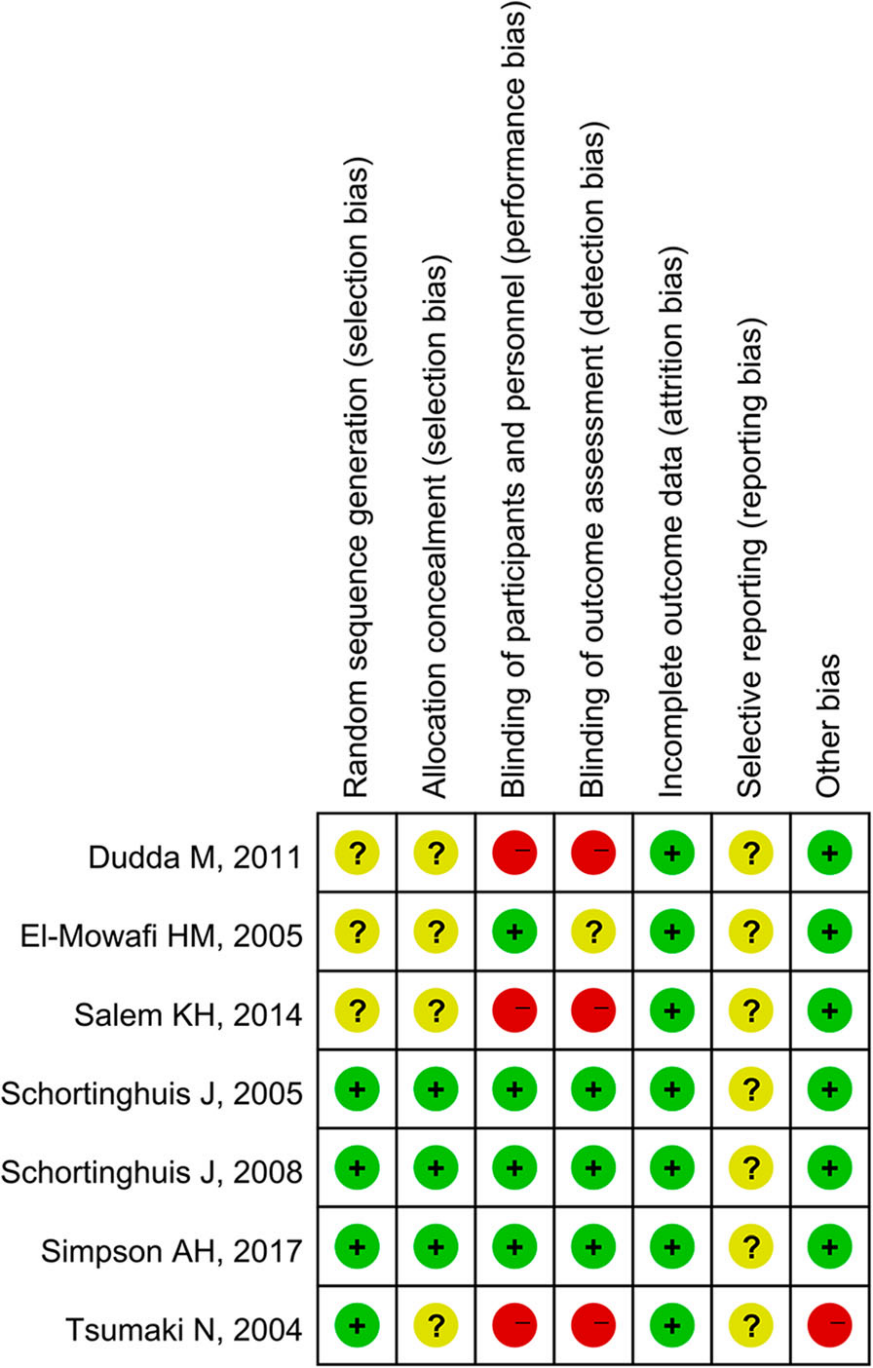
Risk of bias summary. The plus sign means low risk, the question mark means unclear risk, and the minus sign means high risk

Figure 2 summarizes the details of the risk of bias. Overall, we considered 3 trials to be at low risk of bias [20–22], and the other 4 studies to be at high risk of bias [15, 18, 19, 44]. The main limitations were failure to report method for allocation sequence generation [15, 18, 19], allocation concealment [15, 18, 19, 44], unblinded patients [15, 19, 44], and unblinded caregivers or outcome assessors [15, 18, 19, 44]. All the included trials had an unclear risk of reporting bias, because none of the included trials did have a protocol [15, 18–22, 44]. One trial had a potential high risk of other biases, because of the self-control design [44].

### Bone healing index

**Fig. 3:**
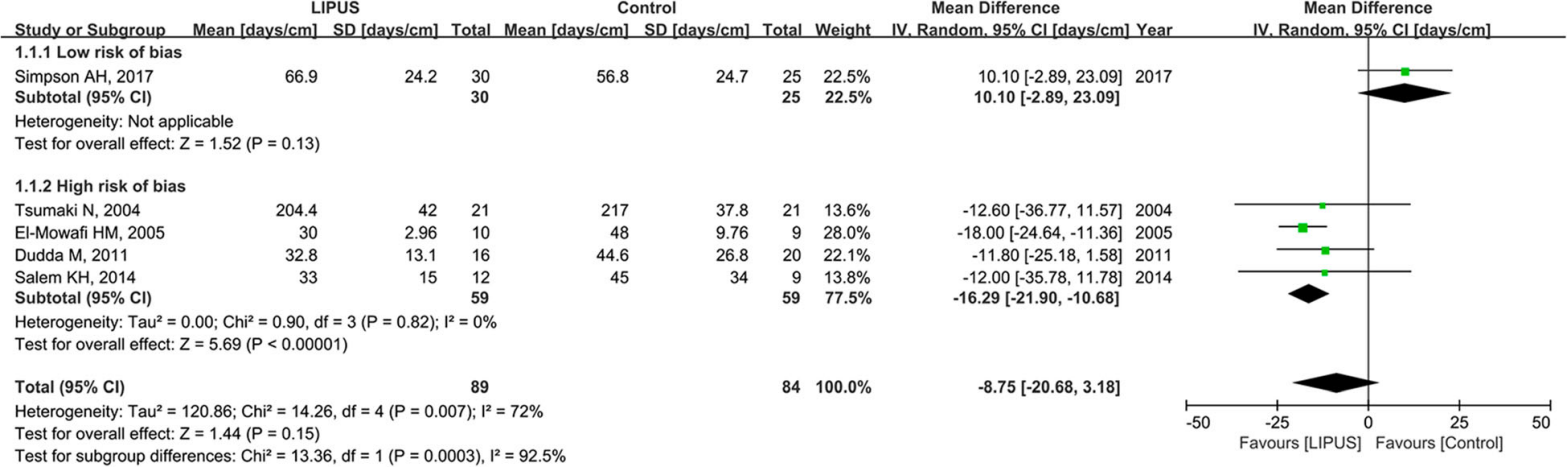
Forest plot for the bone healing index

Five trials, including 152 patients, provided the available data about bone healing index [15, 18, 19, 22, 44]. The analysis did not show a statistically significant reduction in the treatment time in favor of LIPUS (mean difference, − 8.75 days/cm; 95% CI, − 20.68 to 3.18 days/cm; *P* = 0.15; *I*^2^ = 72%; Fig. 3).

Owing to the significant heterogeneity, sensitivity analyses were performed by omitting each study in turn, and the pooled mean difference was directly affected by one trial (Additional file 2: Figure S1) [22]. Subgroup analyses suggested that the combined mean differences were 10.10 days/cm (95% CI, − 2.89 to − 23.09 days/cm; *I*^2^ = 0%) in trials with a low risk of bias and − 16.29 days/cm (95% CI, − 21.90 to − 10.68 days/cm; *I*^2^ = 0%) in trials with a high risk of bias (Fig. 3). The test for subgroup differences indicated that the findings from low risk of bias and high risk of bias subgroups were statistically significantly different from each other (*P* < 0.01 for interaction). The funnel plot suggested there was no significant publication bias (Additional file 3: Figure S2).

### Risk of complications

**Fig. 4:**
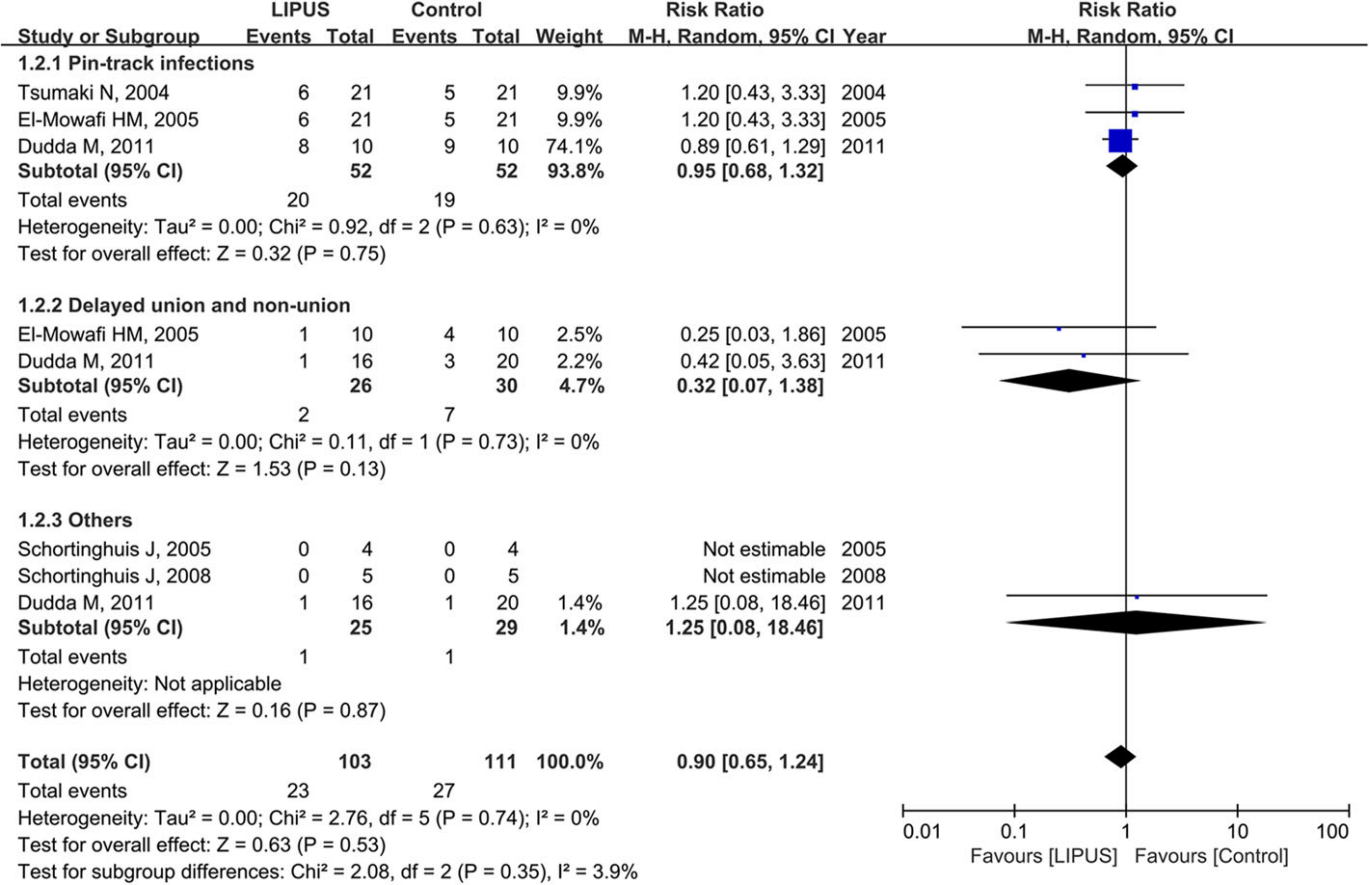
Forest plot for the risk of complications

Five trials reported the incidence rate of complications [15, 18, 20, 21, 44]. Neither the pooled risk ratio (0.90 in favor of LIPUS; 95% CI, 0.65 to 1.24; *I*^2^ = 0%; 3 trials; Fig. 4) nor the pooled risk difference (3% reduction with LIPUS, 13% reduction to 6% increase; *I*^2^ = 0%; 5 trials) showed a significant effect. There was no significant interaction with kinds of complications (risk ratio, *P* = 0.35; risk difference, *P* = 0.39; Fig. 4).

### Other outcomes

**Fig. 5:**
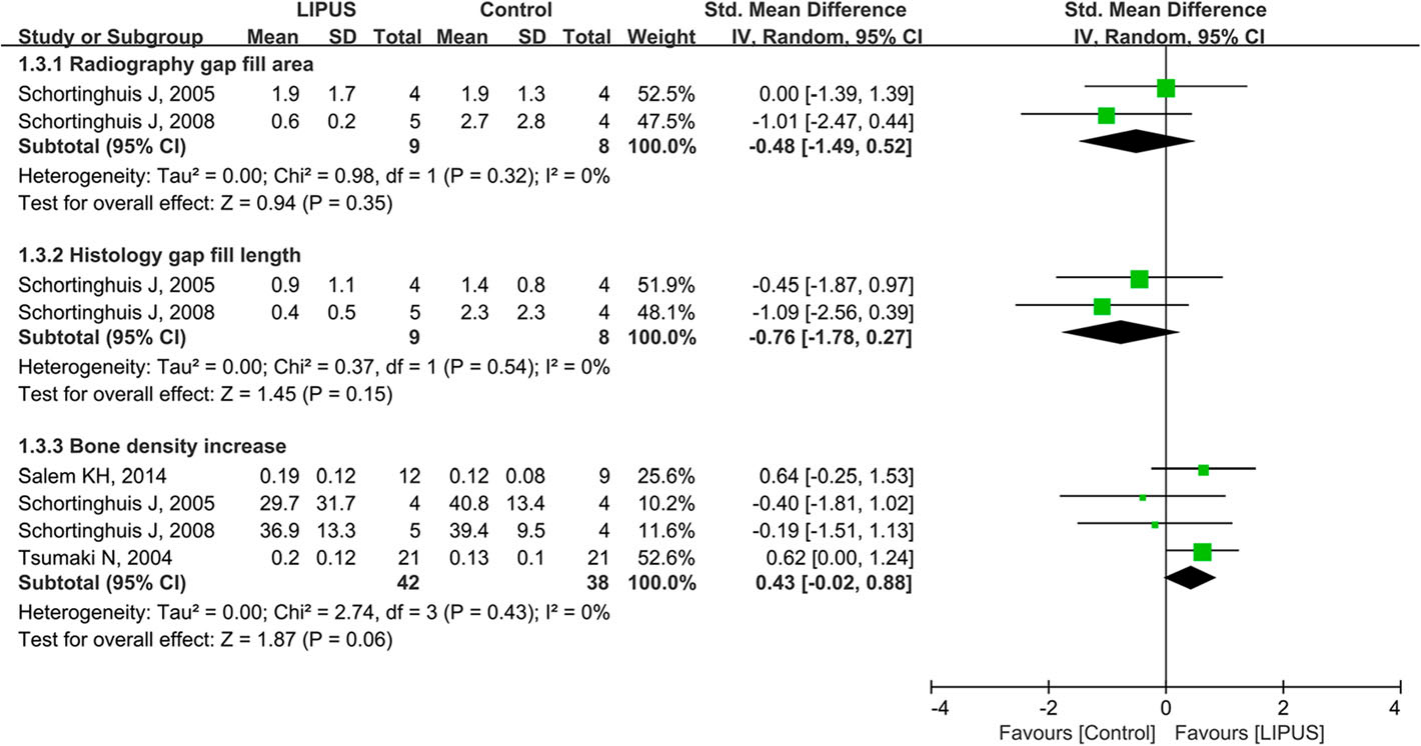
Forest plot for the other outcomes

Four studies reported other outcomes about bone healing [19–21, 44]. Two trials used radiological and histological methods to assess bone regeneration at the distraction gap [20, 21] and showed no significant effect of LIPUS for the radiological gap fill area (standardized mean difference, 0.48 in favor of control; 95% CI, − 1.49 to 0.52; *I*^2^ = 0%) or the histological gap fill length (standardized mean difference, 0.76 in favor of control; 95% CI, − 1.78 to 0.27; *I*^2^ = 0%) (Fig. 5). Four trials assessed bone healing with the bone density increase [19–21, 44], and the overall results suggested no significant effect with LIPUS (standardized mean difference, 0.43 in favor of LIPUS; 95% CI, − 0.02 to 0.88; *I*^2^ = 0%) (Fig. 5).

### Quality of evidence

The GRADE evidence profiles for each outcome are shown in Table 2. All the included trials were RCTs and had no significant publication bias. A risk of bias existed in each outcome except for the outcomes of the radiography gap fill area and the histology gap fill length. Inconsistency existed in the outcome of the bone healing index, which was due to the significant heterogeneity. Imprecision existed in the outcome of the radiography gap fill area, the histology gap fill length, and the bone density increase. Although the included RCTs were considered as high-quality evidence, the available evidence of each outcome was moderate to low, which was downgraded from high due to the above limitations.

**Table 2:** The GRADE evidence quality for each outcome

Quality assessment							No. of patients		Effect		Quality	Importance
No. of studies	Design	Risk of bias	Inconsistency	Indirectness	Imprecision	Other considerations	New comparison	Control	Relative (95% CI)	Absolute		
Bone healing index (better indicated by lower values)												
5	Randomized trials	Serious	Serious	No serious indirectness	No serious imprecision	None	89	84	N/A	MD 8.75 lower (20.68 lower to 3.18 higher)	⊕⊕OO Low	Critical
Risk of complications												
5	Randomized trials	Serious	No serious inconsistency	No serious indirectness	No serious imprecision	None	23/103 (22.3%)	27/111 (24.3%)	RR 0.90 (0.65 to 1.24)	24 fewer per 1000 (from 85 fewer to 58 more)	⊕⊕⊕O Moderate	Critical
Radiography gap fill area (better indicated by higher values)												
2	Randomized trials	No serious risk of bias	No serious inconsistency	No serious indirectness	Very serious	None	9	8	N/A	SMD 0.48 lower (1.49 lower to 0.52 higher)	⊕⊕OO Low	Important
Histology gap fill length (better indicated by higher values)												
2	Randomized trials	No serious risk of bias	No serious inconsistency	No serious indirectness	Very serious	None	9	8	N/A	SMD 0.76 lower (1.78 lower to 0.27 higher)	⊕⊕OO Low	Important
Bone density increase (better indicated by higher values)												
4	Randomized trials	Serious	No serious inconsistency	No serious indirectness	Serious	None	42	38	N/A	SMD 0.43 higher (0.02 lower to 0.88 higher)	⊕⊕OO Low	Important

*N/A* not applicable, *RR* risk ratio, *SMD* standardized mean difference, *MD* mean difference, *CI* confidence interval

⊕⊕⊕⊕ means high qulity

⊕⊕⊕Ο means moderate qulity

⊕⊕ΟΟ means low qulity

⊕ΟΟΟ means very low qulity

## Discussion

### Main findings

Our meta-analysis comprehensively and systematically reviews the current available literature, and the overall results provide low- to moderate-quality evidence that LIPUS applied to patients undergoing distraction osteogenesis has no effect on promoting the process of bone healing or reducing the risk of complications. This study also provides low-quality evidence that LIPUS treatment does not have an advantage of improving the radiological gap fill area, the histological gap fill length, or the bone density increase.

### Comparison with other studies

Our results are inconsistent with the previous systematic reviews, which indicate that LIPUS has a benefit for accelerating healing on distraction osteogenesis [25–29]. Our study differs from previous systematic reviews in several important aspects. First, we include the recently published trial [22], by far the largest trial on LIPUS treatment for distraction osteogenesis. Besides that, we also included one trial [21], which was missed by the previous meta-analysis [29]. Thus, eligible trials in our study were the most comprehensive. Owing to a larger sample size included in our study, we determined a different overall result from the previous meta-analysis. Second, this meta-analysis adds to the existing literature by not only assessing the outcome about bone healing, but also assessing the outcomes about the risk of complications, which is considered as a critical outcome by patients [45]. Based on this advantage, although our subgroup analyses and the previous meta-analysis [29] suggested that LIPUS had a benefit of 16 days/cm, whether LIPUS treatment could be used for all the patients is doubtful. Based on the current evidence [29, 44], a reduction of 16 days/cm, without a reduced risk of complications, might not have a clinical benefit for patients with a small bone defect (< 1 cm). Third, both sensitivity analyses and subgroup analyses were used in our study to explore the heterogeneity, and the heterogeneity of each outcome seems to get a reasonable explanation. Both the sensitivity analyses and the subgroup analyses found that the study by Simpson and colleagues [22] obviously affects the effect of LIPUS, suggesting the study design and/or sample size might be key factors. Finally, we used the GRADE approach to assess the quality of evidence, which was not used by the previous meta-analysis [29]. Since the available evidence of each outcome was only moderate to low, it is suggested that the conclusions of our study may be changed by the future studies.

### Limitations

This study has limitations. First, there were some methodological limitations in the included trials, such as the unclear random method, the inadequate concealment of treatment allocation, and the open-label design. Second, the possibility of publication bias existed. Third, since not all the included trials clearly report the baseline information, it is unclear whether the age, sex, distraction rate, the size of distraction gap, and other variables could affect the effectiveness of LIPUS treatment. Given these limitations, the results of this meta-analysis should be interpreted cautiously.

### Implications for further studies

Since several gaps remain regarding the LIPUS treatment for distraction osteogenesis, future trials are still needed. The design of the future trials should focus on the following points: (1) since subgroup analyses suggest that the results were significantly different between trails at low risk of bias and those at high risk of bias, and all the trials at low risk of bias suggest that LIPUS has no effect for patients undergoing distraction osteogenesis [20–22], trials should pay attention to the methodological design, and double-blinded and clearly reported randomized controlled trials are required, and (2) trials should clearly report the age, sex, and the size of distraction gap to determine whether these variables could affect the effectiveness of LIPUS treatment. Moreover, since smoking, diabetes, and soft tissue can be factors to bone healing [46, 47], the baseline of these key factors need attention; (3) trials should report more outcomes, such as the complications and the functional recovery, which are also important outcomes for patients.

## Conclusion

Among patients undergoing distraction osteogenesis, neither the treatment time nor the complications could be reduced by LIPUS therapy. The currently available evidence is insufficient to support the routine use of this intervention in clinical practice.

## Additional files

Additional file 1: **File S1.** The full search strategies used in MEDLINE, EMBASE, and the Cochrane Library. (DOCX 14 kb)

Additional file 2: **Figure S1.** Sensitivity analysis for the bone healing index. (PNG 265 kb)

Additional file 3: **Figure S2.** Funnel plot for the bone healing index. (PNG 95 kb)

## Abbreviations

CI: Confidence interval
GRADE: Grading of Recommendations Assessment, Development, and Evaluation
LIPUS: Low-intensity pulsed ultrasound
RCT: Randomized controlled trial
RR: Risk ratio
SMD: Standardized mean difference
WMD: Mean difference
