# Supplementary material for: Effect of low-intensity pulsed ultrasound on distraction osteogenesis: a systematic review and meta-analysis of randomized controlled trials
Source: J Orthop Surg Res. 2018 Aug 17;13:205. doi: 10.1186/s13018-018-0907-x (PMC6098620; doi:10.1186/s13018-018-0907-x)
Supplement: Supplementary file 1 — File S1. The full search strategies used in MEDLINE, EMBASE, and the Cochrane Library. (DOCX 14 kb) [file 13018_2018_907_MOESM1_ESM.docx]

**MEDLINE** **(ovid)**

1 exp Ultrasonics/ or exp Ultrasonic Therapy/

2 (ultraso$ or LIPUS or shock wave$ or shockwave$ or ESWT).tw.

3 or/1-2

4 exp Distraction osteogenesis/

5 (distract$ adj5 osteogene$).mp.

6 callotasis.mp.

7 or/4-6

8 3 and 7

9 randomized controlled trial.pt. or randomized.mp. or placebo.mp.

10 8 and 9

**EMBASE (ovid)**

1 exp Ultrasonics/ or exp Ultrasonic Therapy/

2 (ultraso$ or LIPUS or shock wave$ or shockwave$ or ESWT).tw.

3 or/1-2

4 exp Distraction osteogenesis/

5 (distract$ adj5 osteogene$).mp.

6 callotasis.mp.

7 or/4-6

8 3 and 7

9 random:.tw. or placebo:.mp. or double-blind:.tw.

10 8 and 9

**Cochrane Library Databases**

#1 MeSH descriptor: [Ultrasonics] explode all trees

#2 MeSH descriptor: [Ultrasonic Therapy] explode all trees

#3 (ultraso* or LIPUS or shock wave* or shockwave* or ESWT):ti,ab,kw

#4 #1 or #2 or #3

#5 MeSH descriptor: [Osteogenesis, Distraction] explode all trees

#6 distract* near/5 osteogene*

#7 callotasis

#8 #5 or #6 or #7

#9 #4 and #8
