# Supplementary figures and images for: Effect of low-intensity pulsed ultrasound on distraction osteogenesis: a systematic review and meta-analysis of randomized controlled trials
Source: J Orthop Surg Res. 2018 Aug 17;13:205. doi: 10.1186/s13018-018-0907-x (PMC6098620; doi:10.1186/s13018-018-0907-x)

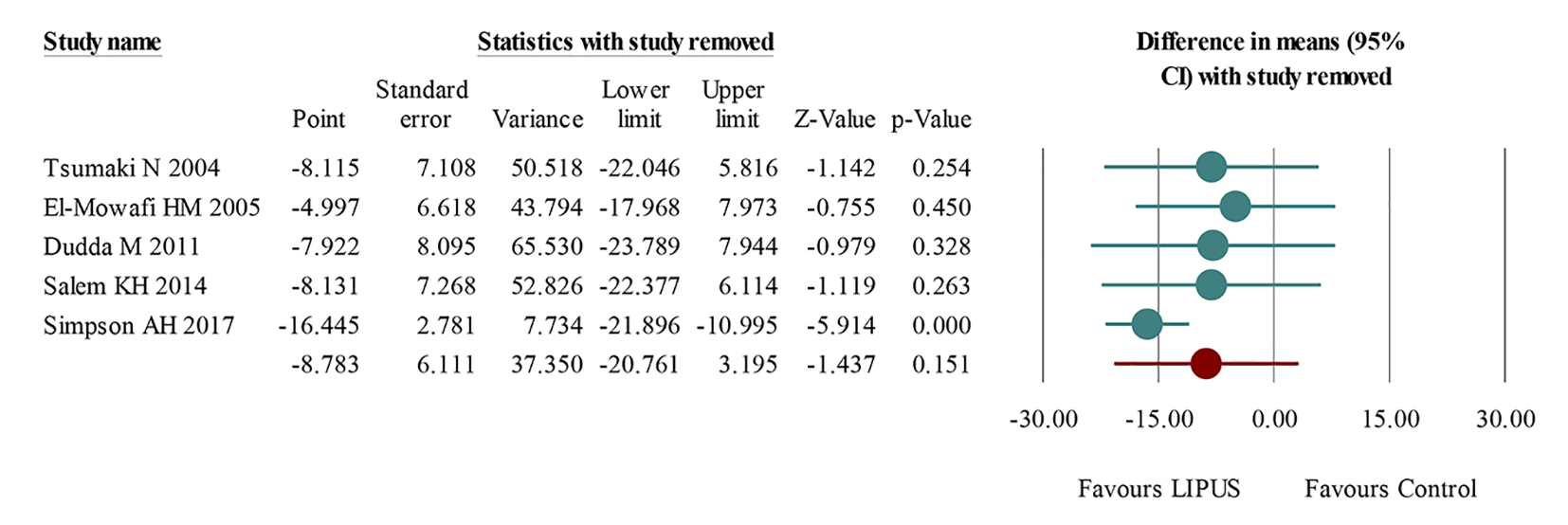

Supplement: Supplementary file 2 — Figure S1. Sensitivity analysis for the bone healing index. (PNG 265 kb) [file 13018_2018_907_MOESM2_ESM.png]

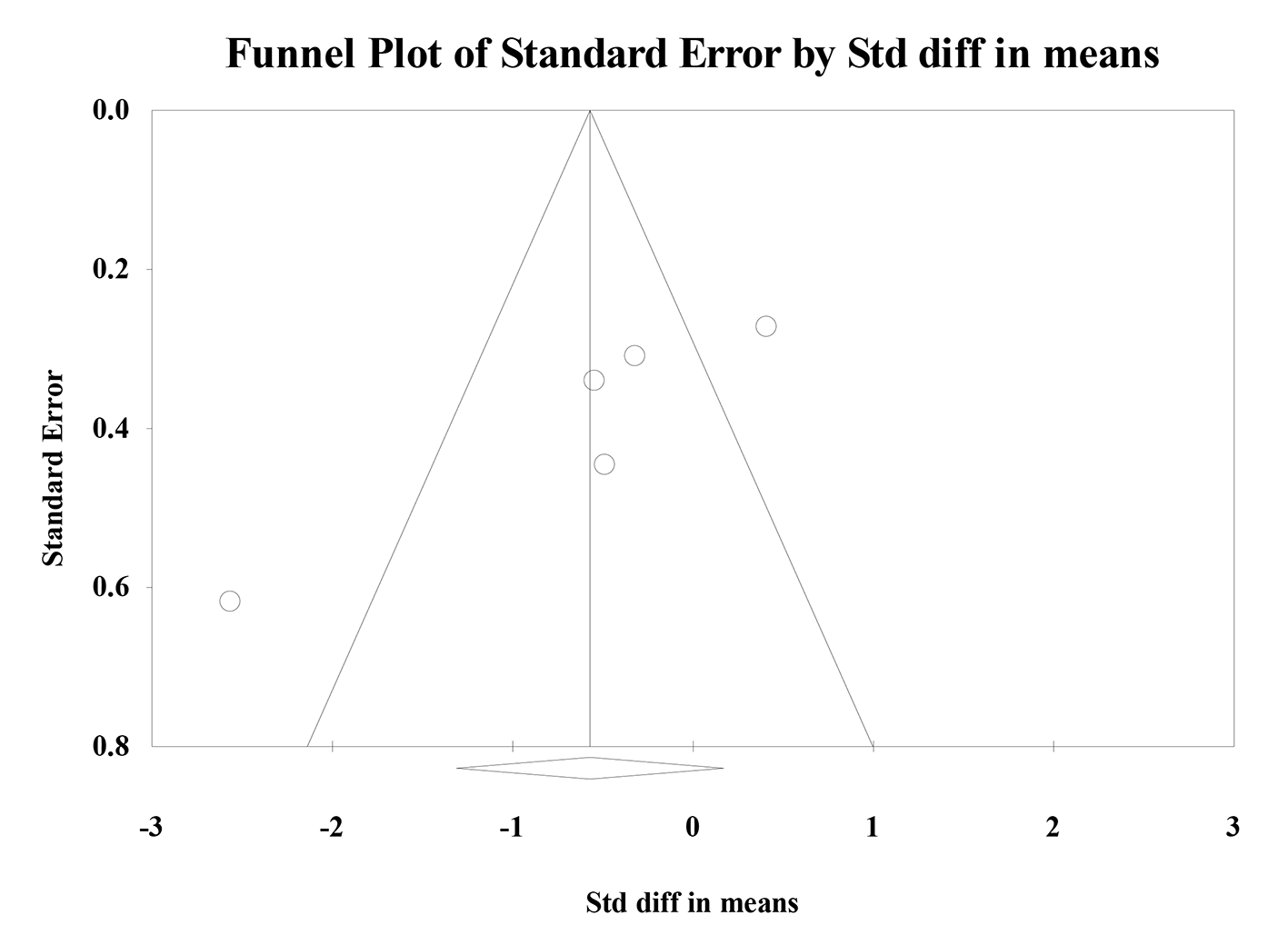

Supplement: Supplementary file 3 — Figure S2. Funnel plot for the bone healing index. (PNG 95 kb) [file 13018_2018_907_MOESM3_ESM.png]
